# Synthesis of Pt_3_Ni Microspheres with High Performance for Rapid Degradation of Organic Dyes

**DOI:** 10.1186/s11671-015-0947-7

**Published:** 2015-05-27

**Authors:** Min Wang, Yushi Yang, Jia Long, Zhou Mao, Tong Qiu, Qingzhi Wu, Xiaohui Chen

**Affiliations:** State Key Laboratory of Advanced Technology for Materials Synthesis and Processing, Wuhan University of Technology, Wuhan, 430070 People’s Republic of China; Department of Prosthetic, School of Stomatology, Wuhan University, Wuhan, 430079 People’s Republic of China

**Keywords:** Pt_3_Ni alloy, Catalytic degradation, Organic dye

## Abstract

In this study, Pt_3_Ni microspheres consisted of nanoparticles were synthesized without addition of surfactants via the solvothermal route. The obtained sample was characterized by X-ray diffraction (XRD), inductively coupled plasma-atomic emission spectrometer (ICP-AES), X-ray photoelectron spectroscopy (XPS), and field-emission scanning electron microscopy (FESEM). Furthermore, the catalytic performance of as-synthesized Pt_3_Ni microspheres was evaluated on the degradation of different organic dyes (methylene blue, methyl orange, Congo red, and rhodamine B). The results show that different dyes were rapidly decomposed by Pt_3_Ni microspheres in different pathways. Among different dyes, the formation and further degradation of the intermediates was observed during the degradation of methylene blue and methyl orange, suggesting the indirect degradation process of these dyes. This study provides not only a promising catalyst for the removal of organic contaminants for environment remediation, but also new insights for Pt_3_Ni alloy as a high-performance catalyst in organic synthesis.

## Background

Organic dyes are typical water pollutants as the by-products of modern textile industry, which result in the breakdown of water ecosystem and greatly threaten the public health because they are potentially carcinogenic and cause various chronic diseases [1–3]. Therefore, the degradation of organic dyes is of great emergency in environment remediation. Various strategies, including physical adsorption [4–6], chemical degradation [7–9], and biodegradation by the microorganisms [10–12], have been developed for decomposing organic dyes from textile effluent. However, these methods are limited with different shortcomings, such as harsh degradation conditions, membrane fouling, low degradation efficiency, and extra post-treatment of sludge [13–16]. PtNi alloy has been proved as a catalyst with high performance for oxygen-reduction reaction [17] and methanol oxidation [18, 19]. Recent studies also demonstrated promising applications of PtNi nanoparticles (NPs) in the detection of glucose from human urine [20], hydrolytic hydrogenation of cellulose [21], catalytic reforming of ethanol, acetone, and methane [22–24]. The exceptionally high catalytic performance of PtNi could be attributed to the reduction of adsorption energy of the reaction intermediates and the increase of active sites on the surfaces of the catalyst when Pt was alloyed with 3d transition metals [25, 26].

In this study, Pt_3_Ni microspheres consisting of NPs were synthesized via a facile solvothermal route without addition of the surfactants. The as-synthesized sample was characterized through X-ray diffraction (XRD), field-emission scanning electron microscopy (FESEM), high-resolution transmission electron microscopy (HRTEM), X-ray photoelectron spectroscopy (XPS), and inductively coupled plasma-atomic emission spectrometer (ICP-AES). Furthermore, the catalytic performance of Pt_3_Ni microspheres was evaluated on the degradation of different dyes, including methylene blue (MB), methyl orange (MO), Congo red (CR), and rhodamine B (Rh-B). The results showed that the Pt_3_Ni microspheres displayed high performance for catalytic degradation of different dyes through an alternative mechanism evidenced by the presence of the intermediates.

## Methods

### Synthesis of Pt_3_Ni microspheres

In a typical synthesis, platinum acetylacetonate (Pt(acac)_2_, 0.25 mmol, Sigma-Aldrich) was added into 20 mL ethylene glycol with magnetic stirring, which was heated to ca. 80 °C to ensure the completed dissolution of platinum salt. Then, NiSO_4_ · 6H_2_O (0.25 mmol, Sinopharm Chemical Reagent Co., Ltd.) was added into another 20 mL ethylene glycol with magnetic stirring. Then, the solution containing platinum salt was added into the NiSO_4_ solution dropwise under magnetic stirring. Subsequently, the mixed solution containing metal salts was transferred into a 50 mL Teflon-lined stainless-steel autoclave and heated to 180 °C for 10 h, and then cooled to room temperature. The precipitate was collected and washed alternately with ethanol and deionized water by centrifugation (9000 rpm, 5 min), and then dried at 60 °C in vacuum.

### Characterizations of Pt_3_Ni microspheres

Phase structure of the as-obtained sample was characterized through X-ray diffraction (XRD, PANalytical X’Pert PRO, Holland) using Cu Kα radiation. The morphology of the sample was observed using a field-emission scanning electron microscopy (FESEM, Hitachi S-4800), and high-resolution transmission electron microscopy (HRTEM, JEM-2100 F STEM/EDS, JEOL Corp, Japan). X-ray photoelectron spectroscopy (XPS) measurement was performed on a VG Multilab 2000 (Thermo Electron Corp., MA) spectrometer using Al Kα radiation as the excitation source. An Optima 4300 DV inductively coupled plasma-atomic emission spectrometer (ICP-AES, Optima 4300DV Perkin-Elmer Corp.) was used to determine the elemental composition of the product.

### Catalytic degradation of different dyes by Pt_3_Ni microspheres

The catalytic performance of Pt_3_Ni microspheres was evaluated by monitoring the degradation of different dyes (MB, 8 mg/L; MO, 20 mg/L; CR, 32 mg/L; and Rh-B, 7 mg/L) using **ultraviolet**–**visible spectroscopy** (UV–vis). In a typical experiment, Pt_3_Ni microspheres (20 mg) was added into 50 mL dye aqueous solutions. The suspension was magnetically stirred at room temperature. The supernatants of the suspensions was collected and centrifuged (12,000 rpm, 5 min) at different time intervals (0, 5, 10, 20, 40, 60, 120, 180, 240 min). Afterwards, the supernatants were collected, and the changes of UV–vis absorption spectra were recorded on a UV–vis spectrophotometer (Shimadzu Corp., UV-2550 PC). In order to determine the degradation capacity of dyes catalyzed by Pt_3_Ni microspheres, the degradation of MB was measured at different concentrations (2, 4, 8, 12, 16 mg/L).

## Results and discussion

**Fig. 1:**
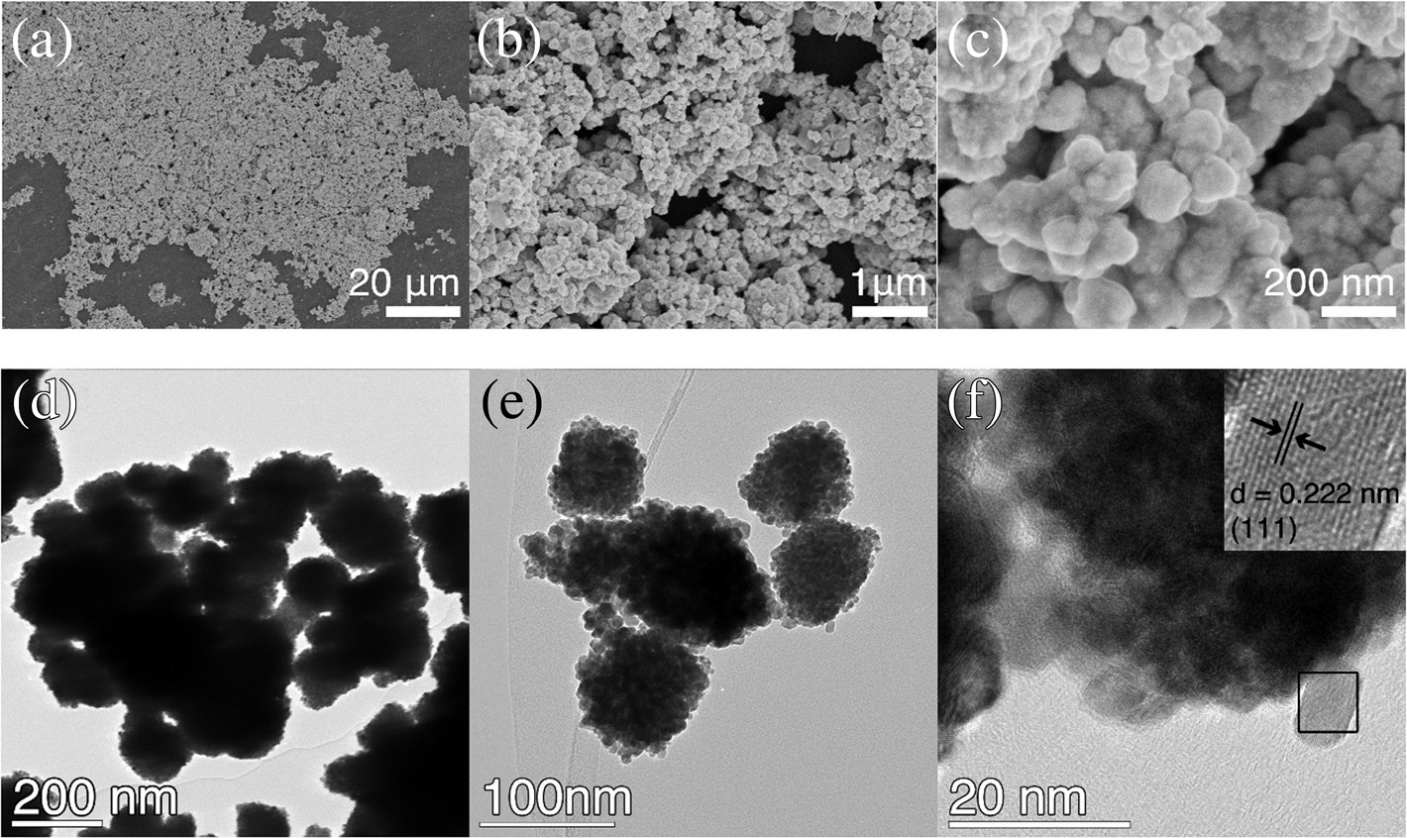
FESEM and TEM images of the as-synthesized sample. **a**–**c** FESEM images of the sample; **d**–**f** TEM images of the sample

Figure 1 shows the FESEM and transmission electron microscope (TEM) images of the sample. As shown in Fig. 1a–c, irregular microspheres were obtained, which consisted of numerous NPs with an average diameter of ca. 50 nm. The assembly of NPs into microspheres was further confirmed by TEM observations (Fig. 1d–f). The well-aligned planes were observed by HRTEM characterization, as shown in the inset in Fig. 1f, indicating the single crystal nature of the as-synthesized sample. The interplanar distance was ca. 0.222 nm, which could be indexed to the adjacent (111) planes of the Pt_3_Ni crystal.

**Fig. 2:**
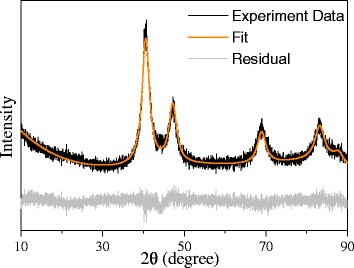
XRD pattern of the as-synthesized Pt_3_Ni microspheres. The *fitted line* and the residual showed the result of the Rietveld refinement. The cell length after refinement was ca. 3.85 Å, lower than that of Pt and larger than that of Ni

Figure 2 shows an XRD pattern of the as-synthesized Pt_3_Ni microspheres. All diffraction peaks could be assigned to a cubic fcc structure. It was noteworthy that all diffraction peaks shifted to a higher 2θ degree compared with those of cubic Pt metal (JCPDS 70–2057), indicating a lower cell length. In order to obtain the cell length, Rietveld refinement was applied to fit the XRD data using Maud software [27]. As shown in Fig. 1, the cell length was ca. 3.850 Å after the refinement, smaller than that of Pt crystal (3.923) and larger than that of Ni crystal (3.523) [28]. This result indicates the incorporation of Ni atoms into the Pt crystal and therefore the formation of PtNi alloy. ICP-AES measurement further confirmed the presence of both Ni and Pt in the sample. The molar/weight percentage of Pt and Ni in the sample was ca. 66.97 and 33.03 %, respectively, suggesting the molar ratio of Pt/Ni was 3/1.

**Fig. 3:**
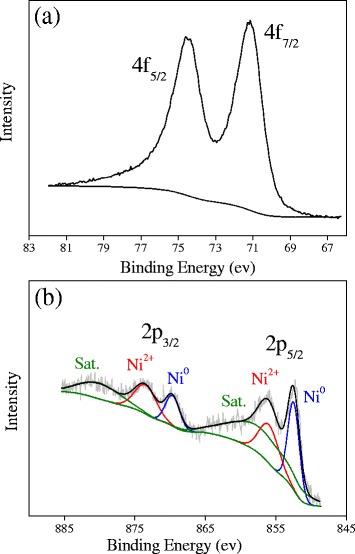
XPS spectra of Pt 4f (**a**) and Ni 2p (**b**) of the as-obtained Pt_3_Ni microspheres

Figure 3 shows the XPS spectra of the as-synthesized Pt_3_Ni microspheres. In Fig. 3a, the fitted peaks at ca. 71.1 and 75.1 eV could be ascribed to 4f_7/2_ and 4f_5/2_ spin orbit of Pt^0^ [18]. The XPS spectrum of Ni was fitted with Gaussian functions by a least-square procedure provided by Numpy and SciPy packages [29]. As shown in Fig. 3b, the fitted peaks at ca. 852, 856, 859 eV and 870, 874, 880 eV could be ascribed to 2p_5/2_ and 2p_3/2_ spin orbit of Ni^0^, Ni^2+^, and their satellite peaks, respectively. The oxidation of Ni might be related to its contact with air. These results further confirmed the formation of Pt_3_Ni alloy.

**Fig. 4:**
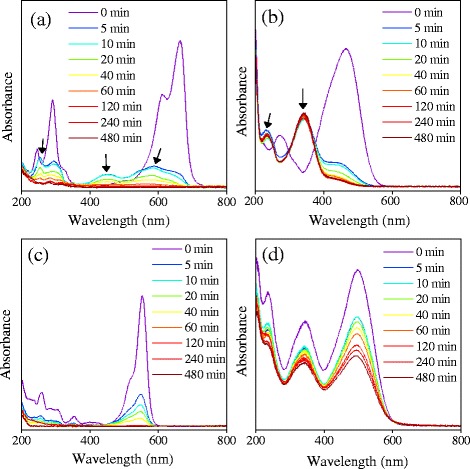
UV–vis spectra of different dye solutions treated with Pt_3_Ni microspheres for different times. **a** MB, 8 mg/L; **b** MO, 20 mg/L; **c** Rh-B, 7 mg/L; **d** CR, 32 mg/L. The absorption peaks of the intermediates were pointed out by the *arrows* in (**a**) and (**b**)

In order to evaluate the catalytic performance of Pt_3_Ni microspheres, the degradation of different dyes was determined by monitoring the changes of UV–vis spectra. Figure 4 shows the UV–vis spectra of different dye solutions (MB, MO, Rh-B, and CR) treated with 20 mg Pt_3_Ni microspheres for different times. It is of great interest that different degradation processes were observed among the four dyes used. As shown in Fig. 4a and b, a series of new absorption peaks appeared immediately in UV–vis spectra of MB and MO solution after 5 min of treatment, indicating the formation of the intermediates. On the other hand, as shown in Fig. 4c and d, the absorption peaks derived from Rh-B and CR were gradually decreased after the treatment with Pt_3_Ni microspheres without the presence of new absorption peaks, suggesting the direct degradation of Rh-B and CR by Pt_3_Ni microspheres.

**Fig. 5:**
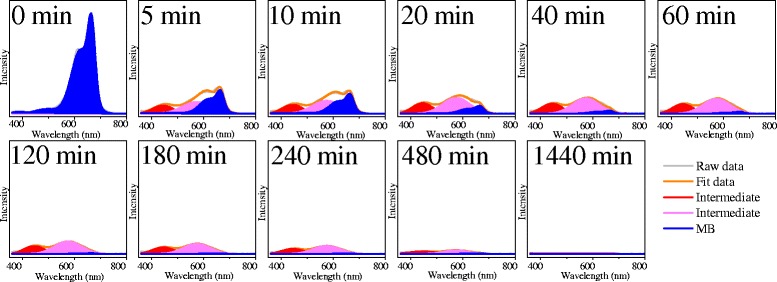
UV–vis spectra fittings of MB solution (16 mg/L) treated with Pt_3_Ni microspheres for different times

In order to explore the relationship between the degradation of MB and the formation of the intermediates, a series of fittings were performed on the UV–vis spectra of MB solution treated with Pt_3_Ni microspheres for different times using a least-square procedure provided by Numpy and SciPy packages [29]. The fittings were set in the wavelength range between 350 to 800 nm for simplification. The spectrum of pure MB solution was firstly fitted with several Gaussian functions. The obtained fitting result represented the profile of MB and was subsequently used for fitting the UV–vis spectra of MB solution treated with Pt_3_Ni microspheres for different times (5, 10, 20, 40, 60, 120, 180, 240, and 1440 min). Figure 5 shows the fitting results of the UV–vis spectra of MB solution at an initial concentration of 16 mg/L. It was obvious that the absorption peaks derived from the intermediates appeared after 5 min of treatment. The absorption intensities derived from the intermediates increased at the initial stage and then decreased gradually. The absorption peaks derived from MB completely vanished after 120 min of treatment, whereas the absorption peaks derived from the intermediates completely vanished after 1440 min of treatment.

The similar fittings were also performed on the UV–vis spectra of MB solutions at different initial concentrations (2, 4, 8, and 12 mg/mL). The degradation of MB and the formation of the intermediates were further compared by relating the absorption intensity with the concentration. The intensity of absorption peaks derived from the intermediates (both the red and blue fitting peak as shown in Fig. 5) was summed and indexed as the concentration of the intermediates.

**Fig. 6:**
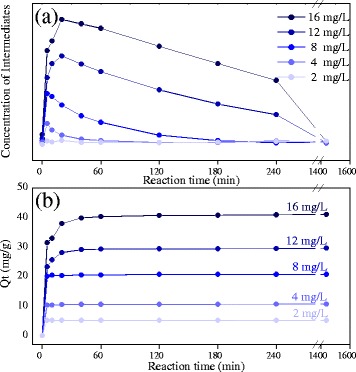
The degradation of intermediates at different initial concentrations of MB. **a** The absorption intensity derived from the intermediates (the *red* and *blue* fitting peak) as shown in Fig. 5 was summed and supposed to be proportional to the concentration of the intermediates; **b** The degradation efficiency (*Q*_*t*_) of the Pt_3_Ni microspheres on MB dye with various initial MB concentrations

Figure 6a shows the formation and degradation of the intermediates at different initial concentrations of MB solution treated by Pt_3_Ni microspheres. It was obvious that the concentration of the intermediates was sharply increased at the initial stage of treatment and subsequently decreased gradually. The completed degradation of the intermediates was dependent on the initial concentration of MB, which finished within 4 h at the MB concentration lower than 8 mg/mL and within 24 h at the MB concentration higher than 12 mg/mL. Figure 6b shows the degradation efficiency (*Q*_*t*_) of the Pt_3_Ni microspheres on MB dye at different initial concentrations. The degradation efficiency of Pt_3_Ni microspheres was calculated as follows:

$$ {Q}_t\kern0.5em =\kern0.5em \left({C}_0\kern0.5em -\kern0.5em {C}_{\mathrm{t}}\right)\kern0.5em *\kern0.5em V/m $$

Where, *C*_*0*_ and *C*_*t*_ are the concentrations of the MB solution at the initial and different time intervals; *V* is the volume of the reaction system (i.e., 50 mL); *m* is the weight of the Pt_3_Ni microspheres used; and *Q*_*t*_ represents the weight of the MB absorbed/degraded per-unit weight of Pt_3_Ni microspheres. At a low initial concentration (2, 4, and 8 mg/mL) of MB, *Q*_*t*_ value was sharply increased and rapidly reached the balance corresponding to the 100 % degradation of MB. However, at a higher initial concentration (12 and 16 mg/mL) of MB, the changes of *Q*_*t*_ value were slower, suggesting that the degradation rate of MB was decreased due to the increase of MB concentration. Therefore, these results implied that the degradation of MB may occur on the surface of Pt_3_Ni NPs.

As observed in the fitted UV–vis spectra, the absorption peaks derived from the intermediates shifted to the shorter wavelength compared with that of MB, implying that the smaller conjunction system contained in the intermediates than that of in MB molecular [30, 31]. In addition, the previous studies also revealed that the aromatic molecules could be adsorbed on the surfaces of both Pt and Ni [32–36]. Therefore, it is speculated that the catalytic degradation of MB molecules involved the adsorption of MB molecules on the surfaces of Pt_3_Ni NPs, the subsequent formation and degradation of the intermediates on the active sites of Pt_3_Ni NPs. More investigations are being undergone to explore the precise formation and degradation mechanisms of the intermediates during the catalytic degradation of MB by Pt_3_Ni NPs.

## Conclusions

In summary, Pt_3_Ni microspheres consisting of nanoparticles were successfully synthesized via a facile route without addition of the surfactants. The as-synthesized Pt_3_Ni microspheres displayed high performance for the catalytic degradation of various organic dyes. The results showed that the degradation of MB and MO by Pt_3_Ni microspheres was involved in the formation of the intermediates. These results not only provide a promising catalyst for the removal of organic contaminants for environment remediation, but also provide new insights for Pt_3_Ni alloy as a high-performance catalyst in organic synthesis.
